# Exploring human papillomavirus vaccination refusal among ethnic minorities in England: A comparative qualitative study

**DOI:** 10.1002/pon.4405

**Published:** 2017-03-15

**Authors:** Alice S. Forster, Lauren Rockliffe, Laura A.V. Marlow, Helen Bedford, Emily McBride, Jo Waller

**Affiliations:** ^1^ Behavioural Science and Health University College London London UK; ^2^ Institute of Child Health University College London London UK

**Keywords:** cancer, England, ethnic minority, HPV vaccine, oncology, qualitative research

## Abstract

**Objectives:**

In England, uptake of human papillomavirus (HPV) vaccination to prevent HPV‐related cancer is lower among girls from ethnic minority backgrounds. We aimed to explore the factors that prevented ethnic minority parents from vaccinating, compared to White British nonvaccinating parents and vaccinating ethnic minority parents.

**Methods:**

Interviews with 33 parents (n = 14 ethnic minority non‐vaccinating, n = 10 White British nonvaccinating, and n = 9 ethnic minority vaccinating) explored parents' reasons for giving or withholding consent for HPV vaccination. Data were analysed using Framework Analysis.

**Results:**

Concerns about the vaccine were raised by all nonvaccinating ethnic minority parents, and they wanted information to address these concerns. External and internal influences affected parents' decisions, as well as parents' perceptions that HPV could be prevented using means other than vaccination. Reasons were not always exclusive to nonvaccinating ethnic minority parents, although some were, including a preference for abstinence from sex before marriage. Only ethnic minority parents wanted information provided via workshops.

**Conclusions:**

Ethnic differences in HPV vaccination uptake may be partly explained by concerns that were only reported by parents from some ethnic groups. Interventions to improve uptake may need to tackle difficult topics like abstinence from sex before marriage, and use a targeted format.

## 1 BACKGROUND

Cancer‐causing types of human papillomavirus (HPV) cause over 99% of cervical cancers.^1^ The virus is spread by skin‐to‐skin contact, including sexual contact. Vaccination against HPV^2^ is offered in England for girls in school year 8 (aged 12‐13) and across the world. Optimal uptake should reduce HPV‐related disease.

Around 89% of eligible girls started the 2‐dose series in England^3^ in 2014/2015, but uptake among girls from ethnic minority backgrounds is lower.^4^, ^5^, ^6^ In England, individuals from ethnic minority groups are generally considered to be those from non‐White British backgrounds, comprising 20% of residents.^7^ Low uptake among ethnic minority groups has been reported across developed countries,^4^ although between‐country comparisons of uptake by specific ethnic groups is complicated by variations in subgroup sizes and migration histories.

Few studies have investigated these ethnic disparities. A small qualitative study with African parents in northern England reported fear of promiscuity and infertility following vaccination, and worry about the vaccine being new.^8^ However, it was not clear how many parents in the study had rejected the vaccine. A qualitative study in southwest England conducted with an ethnically diverse sample not only reported similar findings but also highlighted that language and literacy issues might undermine informed consent,^9^ although this was not limited to ethnic minority families and the majority had received the vaccine. Similar findings have been reported elsewhere, including among African American, Latino, and American Indians in the United States,^10^, ^11^ and Somali mothers in the Netherlands.^12^

Ethnicity is different from race, nationality, religion, and migration status but can include facets of these factors.^13^ Religious beliefs also influence vaccination receipt in minority groups,^14^ independent of ethnicity.^15^ Some ethnic minority mothers have described that some vaccine concerns stemmed from their religious belief, and not their ethnicity,^16^ and a qualitative study with Jewish mothers reported a lack of perceived risk of HPV‐related disease because of circumcision and abstinence from sex before marriage,^17^ a view echoed by Somali mothers in the Netherlands.^12^ However, religious differences in uptake of the vaccine do not always hold across countries.^4^ Migration status and language may also explain uptake.^18^

A limitation of previous research exploring attitudes of individuals from particular subgroups is that it often does not include participants from the population in general, so we do not know if the attitudes expressed are common to the general population and many participants have received the vaccine. We aimed to explore the factors that have prevented parents from ethnic minority backgrounds from vaccinating their daughters against HPV. Secondary aims explored (1) if any of these factors are expressed by nonvaccinating White British parents (suggesting that the factors are not specific to ethnic minority parents) and (2) if any of the factors are expressed by vaccinating ethnic minority parents (suggesting that the factors are not sufficient to stop parents from vaccinating).

## 2 METHODS

Parents of 13‐ to 16‐year‐old girls were recruited through London schools, community groups, online advertising, and through word‐of‐mouth, from 01/03/2015 to 01/03/2016. The focus of this study was nonvaccinating ethnic minority parents. We also recruited a group of vaccinating ethnic minority parents and nonvaccinating White British parents for comparison.

Data were collected via interviews as parents' responses were anticipated to be sensitive. Interviews were audio recorded and transcribed verbatim. Participants provided informed consent. A depth topic guide was used, focusing on participants' experience and opinions about the HPV vaccine. Interviewers took detailed notes following interviews, and occurring themes were discussed between the researchers. Recruitment continued until no new themes were arising. Ethical approval for the study was obtained from the University College London Ethics Committee (3758/001).

Data were analysed using framework analysis, facilitated by NVivo 11 (QSR International Pty Ltd), as it allows comparison of commonalities/differences across participants groups, which was a study aim. Interpretation of the framework was conducted by 3 researchers and discrepancies resolved through discussion.

The results presented are a summary of themes arising from the interviews with nonvaccinating ethnic minority parents. Interviews with vaccinating ethnic minority parents and nonvaccinating White British parents are used to identify where themes/subthemes were exclusive to nonvaccinating ethnic minority parents. Quotes reported are with participant number and ethnicity. Greater methodological detail is available in supplementary material.

## 3 RESULTS

Interviews were conducted with 33 parents, mainly mothers (n = 32), with an average age of 47 years (Table 1). Fourteen parents from ethnic minority backgrounds had not vaccinated their daughters against HPV (4 had daughters who had started the series but not completed it; “partially vaccinated”). Most were from non‐British White or Bangladeshi backgrounds and had a religion. This compares to the London ethnic minority population of whom the largest groups are non‐British White and Black African.^19^ The majority spoke English at home and were born in the UK.

**Table 1:** Sample characteristics

	All Participants (N = 33)	Ethnic Minority Not Vaccinated (n = 14)	Ethnic Minority Vaccinated (n = 10)	White British Not Vaccinated (n = 9)
	n^a^			
Mean age in years (range)	47 (36‐62)	47 (36‐62)	45 (36‐58)	47 (39‐59)
*Religion*				
Christian	10	5	3	2
No religion	8	1	2	5
Muslim	8	4	4	0
Other	6	3	1	2
No response	1	1	0	0
Ethnic group				
White British	9	0	0	9
Non‐British White^b^	8	5	3	0
Bangladeshi	6	3	3	0
African	2	1	1	0
Caribbean	2	1	1	0
Sri Lankan Tamil	1	1	0	0
Somali^c^	1	1	0	0
Indian	1	1	0	0
Pakistani	1	0	1	0
White & Asian	1	1	0	0
White & Black African	1	0	1	0
Main language spoken at home				
English	29	11	9	9
Other	4	3	1	0
Migration status				
Born in UK, as were both parents	10	1	1	8
Born in UK but both parents were not	5	2	3	0
Born in UK but 1 parent was not	4	3	0	1
Not born in UK, neither were parents	13	7	6	0
Not born in UK, neither was 1 parent	1	1	0	0

a Unless specified.

b Including Lithuanian, Italian, Hungarian, South African, American, Austrian, German, and mixed British/Finnish.

c One mother specifically identified herself as Somali and not African.

We also conducted interviews with 10 ethnic minority parents who had vaccinated their daughter and 9 White British parents who had not (1 partially vaccinated; Table 1). See supplementary material for individual participant characteristics (Table S1). All interviews were conducted in English.

### 3.1 Summary of themes

The reasons that ethnic minority parents gave to explain why they had not vaccinated their daughters fit into 4 main themes. First, concerns about the vaccine (1) were raised by all nonvaccinating ethnic minority parents. The 3 other themes overlapped with concerns about the vaccine and included external and internal influences (2), information needs of parents (3), and preventing HPV‐related cancer using means other than vaccination (4). These themes (and their subthemes) were not always exclusive to nonvaccinating ethnic minority parents, although some were (Tables S2‐S4).

### 3.2 Concerns about the vaccine (Table S2)

Vaccinating and nonvaccinating parents expressed a wide range of concerns about the vaccine, suggesting that holding some concerns was not sufficient to stop vaccination.

#### 3.2.1 Concern about side effects

Nonvaccinating and partially vaccinating parents from various ethnic backgrounds expressed concerns about the research behind the vaccine, which made parents worry about potential for side effects.

… the only thing that worries me is … that we don't know the long‐term effects … that's the only worry I have (P27, Bangladeshi).

A nonvaccinating Bangladeshi mother and Indian father were wary of vaccines because they had perceived vaccination to be unsafe or painful when they were children. One partially vaccinating non‐British White mother perceived that her daughter experienced an allergic reaction after the first dose.

Other concerns about side effects reported by a small number of nonvaccinating parents included concern about the potential for vaccine ingredients to cause adverse reactions, concern that their daughter's immune system was compromised and concern that their daughter was being used “like a guinea pig.”

[my daughter] was asthmatic … her symptoms started to worsen a little bit … and I think the second dose was one or two months after this … I just couldn't psychologically take it, so I thought, err, you know, with her symptoms, with her immune system, I'd rather not go ahead (P16, non‐British White).

Four parents, spread across all groups, described feeling as though their child's interests were not being considered. Parents in all groups recognised the potential for side effects in all medicines.

#### 3.2.2 Concerns relating to perceptions of risk

Parents in all groups mentioned that the vaccine was not available when they were younger. This made ethnic minority mothers feel the vaccine was unnecessary as they had been fine without it.

A number of nonvaccinating ethnic minority parents believed their daughters were not at risk of contracting HPV or developing cervical cancer, either because they did not believe the virus/disease to be prevalent or because they had no family history of cervical 0cancer. Others felt that the benefits of vaccination did not outweigh the risks.

Some nonvaccinating parents from Somali, Bangladeshi, mixed Asian, and White British backgrounds perceived their daughters to be at low risk as they would not be promiscuous or have unprotected sex. Nonvaccinating and vaccinating ethnic minority parents felt that 12 to 13 years of age was young to vaccinate against a sexually transmitted infection.

I know what my daughter is like … she goes to a girls' school, she doesn't hang out with boys … She's not of a nature that I think she would naturally be very promiscuous at a young age … I don't look at her and think, okay, she needs to have this (P15, White and Asian).

#### 3.2.3 Concern that the vaccine will promote promiscuity

A number of nonvaccinating ethnic minority and White British parents felt concerned that HPV vaccination would encourage unsafe sexual practices. One Indian father felt the vaccine adverts promoted casual sex.

… I really object to the adverts … that say you can't stop your daughter from growing up, but you can … reduce the risks of her getting cancer …. It's sending the wrong messages. It's claiming that, um, having sex is part of growing up. I don't believe it is (P21, Indian).

#### 3.2.4 Concerns about the effectiveness of the vaccine

A number of nonvaccinating ethnic minority parents were concerned that the vaccine does not protect against all HPV types. One nonvaccinating non‐British White parent and 3 nonvaccinating White British parents felt the duration of vaccine protection was limited.

#### 3.2.5 Concern about motivations behind introducing the vaccine

Nonvaccinating ethnic minority and White British parents believed the vaccine was introduced to make money for pharmaceutical companies and expressed lack of faith in the government, the “system,” and medical professionals.

### 3.3 External and internal influences (Table S3)

#### 3.3.1 Others providing information

Vaccinating and nonvaccinating ethnic minority parents sourced information regarding vaccine side‐effects from others, suggesting that these discussions were not sufficient to stop parents from consenting. Another parent was told that the number of people getting cervical cancer is “very few and far between” (P17, African).

I asked my friend … because I was new in the UK … “What is this?” She says, “Yes, these are the side effects” (P25, Somali).

A non‐British White mother and a White British mother, neither of whom had vaccinated their daughters, described that their friends (a school nurse and a scientist) did not trust the vaccine's development.

#### 3.3.2 Experience of others

A number of nonvaccinating ethnic minority (n = 3) and White British (n = 4) parents made reference to other girls they knew in person or online, who had become unwell after receiving the vaccine. More specifically, parents from mixed Asian (n = 1), Caribbean (n = 1) and non‐British White (n = 2) backgrounds talked about a girl who was reported to have died because of the vaccine in 2009 (although this was later confirmed untrue). Irrespective of vaccination status or ethnicity, a large number of parents spoke about others who had not had the vaccine.

… there was a girl that died. She had it and then she went into hospital …. She died, and then they traced it back to this vaccination. So that, I suppose, has put me off a bit as well (P2, Caribbean).

Three nonvaccinating mothers said that their daughter did not want the vaccination and 1 nonvaccinating Somali mother was advised by a health professional not to vaccinate unless her daughter was going to be “doing a lot of sex” (P25, Somali).

#### 3.3.3 Influence of emotion

Some ethnic minority parents reassured themselves of their decision to not vaccinate by believing that future events are uncertain. Two nonvaccinating mothers anticipated that they would regret vaccinating because they felt there was a risk to their daughters' health. Some ethnic minority parents lacked an emotional connection to their decision.

I haven't got any sort of emotional connection to it, to be honest. I don't feel any way. I just feel like, um, she'll be fine. Life goes on (P2, Caribbean).

### 3.4 Information needs of parents (Table S4)

Almost half of parents (n = 13), regardless of ethnicity or vaccination status, had not heard of the vaccine before being invited to vaccinate their daughters, although a few (n = 8) had.

I didn't know anything much … Err, and still don't know anything about it, really. So I didn't sign the form for her to have it … (P2, Caribbean).

#### 3.4.1 Information requirements

Linked to parents' concerns about safety, many felt insufficiently informed of the side effects from vaccine information they were provided with. Other safety information requested by nonvaccinating parents included information on trials and where in the world the vaccine is offered.

… if you get medication … there's this huge list of side‐effects that's listed in the packet … I don't feel the same degree of care was provided around this …. There was no information provided about the potential side‐effects …. You couldn't possibly make an informed decision from that (P21, Indian).

Some nonvaccinating parents wanted other information about the vaccine, including what it protects against, the duration of efficacy, and on the impact of the vaccine so far. A few wanted to know why the vaccine is given at 12 to 13, and a partially vaccinating mother did not know how many doses are required. Other nonvaccinating parents requested further information about the vaccine in general, including statistics, “enough to convince them,” and sufficient information to make an informed decision. Some ethnic minority parents, including those who had vaccinated their daughters, wanted to speak to “an expert,” although it was not specified who this might be, and to have education workshops where they could ask questions.

… if they said, “Right, I'm holding a night. If you want to know about the injection … come down.” I would be down there and I would take 15 people with me …. I've got twelve godchildren. I would take all seven girls (P17, African).

#### 3.4.2 Further research

Parents differed in the extent that they had done further research into the vaccine; only ethnic minority parents said that they did very little, almost exclusively parents who had vaccinated their daughter, but 3 nonvaccinating ethnic minority parents did no further research because their mind was made up from the start. Other parents, mainly those who had not vaccinated against HPV, described doing a lot of research because they felt ill‐informed by the NHS information. Others did research to confirm their decision not to vaccinate and some said they always question things.

### 3.5 Preventing HPV‐related cancer using means other than vaccination (Table S4)

#### 3.5.1 Illness prevention informed by complementary and alternative medicine and idiosyncratic beliefs

Some, mainly nonvaccinating, parents subscribed to schools of thought relating to HPV vaccination that were not consistent with the standard medical model. Some of these beliefs related to complementary and alternative medicine, while others were more idiosyncratic (although may have been held by many people). Two nonvaccinating ethnic minority parents and 1 vaccinating ethnic minority parent mentioned a preference to not use medicines in general, one of whom had never given their child vaccines.

Many parents used methods to prevent illness that were based on these idiosyncratic beliefs, including building immunity “naturally” and preventing cancer/illness with a healthy lifestyle. Almost exclusively, it was ethnic minority parents who had never vaccinated their children who reported using complementary and alternative medicine.

… as long as we lead a healthy lifestyle without any bad habits … I should be able to protect myself from any kind of illness. Like … cancer and stuff like that (P29, Bangladeshi).

#### 3.5.2 Preventing cervical cancer using approaches other than vaccination

Many parents who had not vaccinated their daughter explained that they would prefer to use approaches other than vaccination to prevent cervical cancer. These included encouraging safe sex and education/discussion and were mainly mentioned by nonvaccinating ethnic minority parents.

… if we want to avoid HPV, let's go out there and use condoms …. It's … good sexual dialogue with your teenagers. I think that's more important for them than having a vaccination … (P22, non‐British White).

Only nonvaccinating ethnic minority parents (n = 5) discussed abstinence from sex before marriage. Sometimes this was discussed in relation to religion by parents who were all practising Muslims. Two nonvaccinating ethnic minority parents also said that they would encourage their daughter not to be promiscuous.

I always give her advice. “Your body is your body and you will sleep with only one man … if you like someone, just tell him, ‘Wait for me in the future …’” (P25, Somali).

#### 3.5.3 External forces

Two nonvaccinating ethnic minority parents believed that cancer is controlled by fate or God, which may have influenced their lack of perceived need for vaccination.

… what I believe cervical cancer is … if you get that, it's a gift from the God (P25, Somali).

## 4 DISCUSSION

Our analysis of interviews conducted with nonvaccinating ethnic minority parents identified that parents' reasons for not vaccinating their daughter against HPV related to 4 themes. Interviews with nonvaccinating White British parents and ethnic minority vaccinating parents allowed insight into whether the reasons given by nonvaccinating ethnic minority parents were only mentioned by parents from some ethnic groups and/or were sufficient to stop vaccination.

Nonvaccinating ethnic minority parents reassured themselves of their decision by reporting that there are approaches other than vaccination to protect against HPV, including abstinence from sex before marriage, which was often related to religious beliefs. It is known that some religious beliefs are not conducive to vaccination.^20^ Similar reasoning has been reported elsewhere in the UK and in other developed countries.^8^, ^12^, ^21^, ^22^ Other parents were apathetic about the disease, because they were unvaccinated themselves and had not developed cervical cancer.

While there were reasons for not vaccinating that were only reported by parents from some ethnic minority groups, many reasons were cited by parents regardless of ethnic background and were reflective of the concerns that parents have expressed about vaccines in general.^14^ Previous research has suggested that ethnic minority parents are more likely to hold negative beliefs than White British parents.^15^ This, along with the present study, suggests that ethnic disparities in uptake may be explained both by differences in the proportion of people from different ethnic groups holding negative beliefs about the vaccine and beliefs that are only held by parents from some ethnic minority groups. This has not been explored elsewhere, and it would be interesting to examine if this hypothesis is substantiated in other countries.

Reasons for not vaccinating identified in this study could be used to guide the development of interventions to increase uptake of HPV vaccination. However, international evidence is lacking that changing beliefs/education influences vaccination,^23^ suggesting that a different approach is needed. In the English immunisation programme, parents should receive a 2‐page leaflet when asked to consent to their daughter receiving the vaccine, containing signposting to further information, including a detailed leaflet.^24^ In our study, only parents from ethnic minority backgrounds requested that information be provided via workshops, suggesting that the format of information needs to be tailored, although changing the content may not improve uptake.

Parents from some ethnic groups are accurate in stating that HPV‐related disease is rare in their communities, but this may be changing. Asian women in the UK are at reduced risk of getting cervical cancer compared to White women,^25^ which is assumed to be due to sexual practices. Cervical cancer rates are also lowest among Asian/Pacific Islander women in the United States;^26^ although between country, comparisons are difficult due to varying countries of origin within this ethnic subgroup and migration history. However, daughters may not choose the same lifestyle as their parents. Protection against HPV without vaccination is also dependent on sexual partners being monogamous for life. In the UK, almost 50% of men from Pakistani and Indian backgrounds, where it is traditional to abstain from sex before marriage, have had sex with more than 1 partner,^27^ so unvaccinated women from these communities may still be at risk, even if they abstain from premarital sex. This will be a difficult message to communicate to some parents and may need input from community leaders.

### 4.1 Study limitations

Cost prohibited translating the recruitment materials into all languages spoken in London. No one approached the study team as a result of translated materials or asked to conduct the study in Somali or Bengali, but there remains the possibility that we were not able to access non‐English speaking/reading individuals. Only 0.6% of London residents do not speak English.^28^ It is unlikely that this small population, many of whom will not have daughters eligible for vaccination, will make a huge impact on vaccination coverage. Only 1 father participated, which may have been because the recruitment materials were more likely to have been received by the mother; most participants were recruited via correspondence sent via their daughter's school. However, every mother described that the decision was made by her or was shared. A minority of interviews (n = 7) were conducted via telephone. It is possible that information was withheld or was more likely to have been given using this different approach to data collection, although this was not obviously apparent.

### 4.2 Clinical implications

Ethnic disparities in uptake of HPV vaccination in England may in part be explained by concerns that were only reported by parents from some ethnic minority groups, as well as differences in the prevalence of concerns about the vaccine that are held by nonvaccinating parents in general. Interventions to increase uptake of the vaccine will need to take a different approach to those that have been tested to date. Such interventions may need to tackle difficult topics like abstinence from sex before marriage, as well as using a targeted format.

## CONFLICT OF INTEREST

The authors have declared no conflicts of interest.

## Supporting information

Data S1 Supporting info item
