# Supplementary material for: Exploring human papillomavirus vaccination refusal among ethnic minorities in England: A comparative qualitative study
Source: Psychooncology. 2017 Mar 15;26(9):1278–84. doi: 10.1002/pon.4405 (PMC5599953; doi:10.1002/pon.4405)
Supplement: Supplementary file 1 — Data S1 Supporting info item [file PON-26-1278-s001.docx]

**Supplementary material – additional methodological detail**

Parents of 13-16 year old girls were recruited through London schools, community groups, online advertising and through word-of-mouth, from 01/03/2015-01/03/2016. The focus of this study was non-vaccinating ethnic minority parents. We also recruited a group of vaccinating ethnic minority parents and non-vaccinating White British parents for comparison. Ethnicity was self-reported. Recruitment materials were translated into Somali and Bengali. Translators were available for interviews and recruitment. It is known that uptake of the HPV vaccine is lower among girls from Black and Asian backgrounds [[1-3](#_ENREF_1)], so we translated the materials into one language spoken by individuals from each group. We chose Somali and Bengali as they are spoken by significant sized populations in London and among those who do not speak English [[4](#_ENREF_4), [5](#_ENREF_5)].

Data were collected via interviews as it was anticipated that parents’ responses may be of a sensitive nature. Parents were not given the opportunity to contribute data other than via interviews. Interviews were conducted by one of two female researchers either at the participants’ home or place of work (n=26) or on the telephone (n=7). They lasted an average of 38 minutes, were audio-recorded and transcribed verbatim. Participants provided informed consent. A depth topic guide was used, focusing on participants’ experience of childhood vaccinations in general, and their experience and opinions about the HPV vaccine. Interviewers took detailed notes after each interview and occurring themes were discussed between the researchers. Recruitment continued until the research team felt that data saturation had occurred (no new themes were arising). Ethical approval for the study was obtained from the University College London Research Ethics Committee (3758/001).

Data were analysed using Framework Analysis, facilitated by NVivo 11 (QSR International Pty Ltd), as it allows analysts to compare commonalities and differences in themes across participants groups, which was an aim of this study. After familiarisation with the data by all analysts, transcripts were independently coded line-by-line by two researchers and codes refined to create sub-themes and major themes by three researchers. Transcripts were then entered into a matrix that organised participant quotes according to code/theme and participant characteristics (ethnic minority non-vaccinators, ethnic minority vaccinators and White British non-vaccinators), allowing for comparison of quotes between groups. Interpretation of this matrix was conducted by three researchers and discrepancies resolved through discussion.

The results presented are a summary of the themes arising from the interviews with ethnic minority parents who had not vaccinated their daughter. Interviews with parents from ethnic minority backgrounds who had vaccinated and White British parents who had not are used to identify where themes/sub-themes were exclusive to non-vaccinating ethnic minority parents. Quotes reported are those said by non-vaccinating ethnic minority parents, with participant number and self-reported ethnicity. Where ‘Mixed Asian’ or ‘Mixed Black African’ is used, participants had reported that they also were of White ethnicity (e.g. White and Asian).

**References**

1. Roberts SA, Brabin L, Stretch R, Baxter D, Elton P, Kitchener H, McCann R: **Human papillomavirus vaccination and social inequality: results from a prospective cohort study**. *Epidemiology and infection* 2011, **139**(3):400-405.

2. Fisher H, Audrey S, Mytton JA, Hickman M, Trotter C: **Examining inequalities in the uptake of the school-based HPV vaccination programme in England: a retrospective cohort study**. *J Public Health (Oxf)* 2013, **36**(1):36-45.

3. Brabin L, Roberts SA, Stretch R, Baxter D, Chambers G, Kitchener H, McCann R: **Uptake of first two doses of human papillomavirus vaccine by adolescent schoolgirls in Manchester: prospective cohort study**. *Bmj* 2008, **336**(7652):1056-1058.

4. Gopal D, Matras Y: **Who can and cannot speak English**. In: *Dynamics of diversity: evidence from the 2011 census.* Edited by (CoDE) ECoDoE. Manchester: University of Manchester; 2011.

5. Krausova A, Vargas-Silva C: **London: Census Profile**. In*.* Oxford: University of Oxford; 2013.

**Supplementary material Table 1 (SM1) – Individual participant characteristics**

| **No.** | **Daughter’s HPV vaccine status** | **Ethnic group** | **Religion** | **Main language spoken at home** | **Migration status** |
| --- | --- | --- | --- | --- | --- |
| 1 | Vaccinated | Bangladeshi | Muslim | English/Bengali | Born in UK but both parents were not |
| 2 | Not vaccinated | Caribbean | Christian | English | Not born in UK, neither were parents |
| 3 | Vaccinated | African | Christian | English/Yoruba | Born in UK but both parents were not |
| 4 | Vaccinated | Bangladeshi | Muslim | English/Bengali | Not born in UK, neither were parents |
| 5 | Vaccinated | Bangladeshi | Muslim | Bangla | Not born in UK, neither were parents |
| 6 | Vaccinated | Pakistani | Muslim | English | Born in UK but both parents were not |
| 7 | Partially vaccinated | Sri Lankan Tamil | Hindu | Tamil | Not born in UK, neither were parents |
| 8 | Not vaccinated | Non-British White | Christian | Lithuanian | Not born in UK, neither were parents |
| 9 | Vaccinated | White & Black African | Unitarian | English | Not born in UK, neither were parents |
| 10 | Vaccinated | Non-British White | No religion | English | Not born in UK, neither were parents |
| 11 | Vaccinated | Caribbean | Christian | English | Born in UK, as were both parents |
| 12 | Vaccinated | Non-British White | Christian | English | Not born in UK, neither were parents |
| 13 | Vaccinated | Non-British White | No religion | English | Not born in UK, neither were parents |
| 14 | Not vaccinated | White British | No religion | English | Born in UK, as were both parents |
| 15 | Not vaccinated | White & Asian | Christian | English | Born in UK but one parent was not |
| 16 | Partially vaccinated | Non-British White | Christian | English | Not born in UK, neither were parents |

| **No.** | **Daughter’s HPV vaccine status** | **Ethnic group** | **Religion** | **Main language spoken at home** | **Migration status** |
| --- | --- | --- | --- | --- | --- |
| 17 | Not vaccinated | African | Christian | English | Born in UK but one parent was not |
| 18 | Not vaccinated | White British | Jewish | English | Born in UK, as were both parents |
| 19 | Not vaccinated | White British | No religion | English | Born in UK, as were both parents |
| 20 | Partially vaccinated | White British | No religion | English | Born in UK, as were both parents |
| 21 | Not vaccinated | Indian | Other | English | Not born in UK, neither was one parent |
| 22 | Not vaccinated | Non-British White | Lutheran | English | Born in UK but one parent was not |
| 23 | Not vaccinated | White British | No religion | English | Born in UK, as were both parents |
| 24 | Partially vaccinated | Non-British White | Not given | English | Not born in UK, neither were parents |
| 25 | Not vaccinated | Somali | Muslim | Somali/Arabic/ Swedish/English | Not born in UK, neither were parents |
| 26 | Not vaccinated | White British | Christian | English | Born in UK, as were both parents |
| 27 | Not vaccinated | Bangladeshi | Muslim | English | Born in UK but both parents were not |
| 28 | Not vaccinated | White British | No religion | English | Born in UK, as were both parents |
| 29 | Not vaccinated | Bangladeshi | Muslim | Bengali | Born in UK but both parents were not |
| 30 | Not vaccinated | White British | Christian | English | Born in UK but one parent was not |
| 31 | Not vaccinated | White British | Jewish | English | Born in UK, as were both parents |
| 32 | Partially vaccinated | Non-British White | No religion | English | Not born in UK, neither were parents |
| 33 | Not vaccinated | Bangladeshi | Muslim | English | Born in UK, as were both parents |

**Supplementary material table 2 (SM2) – Sub-themes of Theme 1 and whether they were mentioned by ethnicity / vaccination status**

| **Theme 1 - Concerns about the vaccine** | **Ethnic minority not vaccinated** | **Ethnic minority vaccinated** | **White British not vaccinated** |
| --- | --- | --- | --- |
| **Concern about side-effects** |  |  |  |
| Concerns about research supporting use of the vaccine | 5 |  | 7 |
| Previous bad experience of vaccinations | 3 |  |  |
| Concern about vaccine ingredients | 3 |  | 2 |
| Perceive daughter’s immune system is compromised | 2 |  |  |
| Girls are being used as guinea pigs | 1 |  | 1 |
| Daughter not being treated as an individual | 2 | 1 | 1 |
| All medicines have potential for side-effects | 3 | 3 | 1 |
| **Concerns relating to perceptions of risk** |  |  |  |
| Vaccine was not available when parent was younger | 2 | 1 | 2 |
| Do not perceive daughter to be at risk | 5 |  | 2 |
| Risks of vaccination outweigh the benefits | 4 |  | 4 |
| Not necessary to vaccinate against HPV at 12-13 years | 3 | 1 |  |
| **Concern vaccination will promote promiscuity** |  |  |  |
| Concern vaccination will promote promiscuity | 5 |  | 2 |
| **Concerns about the effectiveness of the vaccine** |  |  |  |
| The vaccine does not protect against all HPV types | 3 |  |  |
| Concern about duration of protection | 1 |  | 3 |
| **Concern about motivations behind introducing the vaccine** |  |  |  |
| Believe that the vaccine is a money making exercise | 3 |  | 2 |
| Lack of faith in government’s approach to vaccination | 2 |  | 2 |
| Lack of trust in the ‘system’ | 1 |  |  |
| Lack of trust in medical profession | 1 |  | 1 |

*Note: n* denotes number of participants who mentioned the sub-theme**Supplementary material table 3 (SM3) – Sub-themes of Theme 2 and whether they were mentioned by ethnicity / vaccination status**

| **Theme 2 – External and internal influences** | **Ethnic minority not vaccinated** | **Ethnic minority vaccinated** | **White British not vaccinated** |
| --- | --- | --- | --- |
| **Others providing information** |  |  |  |
| Informed about side-effect by others | 2 | 1 |  |
| Told that cervical cancer is rare | 1 |  |  |
| Told that the vaccine research is unreliable | 1 |  | 1 |
| **Experience of others** |  |  |  |
| Perceive that other girls experienced side effects | 3 |  | 4 |
| Reports of a girl who had died following HPV vaccination | 1 | 2 |  |
| Knowing others who have not received the vaccine | 6 | 1 | 3 |
| **Daughter did not want the vaccine** |  |  |  |
| Daughter did not want the vaccine | 2 |  | 1 |
| **Advised not to vaccinate** |  |  |  |
| Advised not to vaccinate | 1 |  |  |
| **Influence of emotion** |  |  |  |
| Uncertainty about the future | 3 |  |  |
| Anticipated regret | 1 |  | 1 |
| Emotional disengagement | 3 | 3 |  |

**Supplementary material table 4 (SM4) – Sub-themes of Themes 3-4 and whether they were mentioned by ethnicity / vaccination status**

| **Theme 3 - The information needs of parents** | **Ethnic minority not vaccinated** | **Ethnic minority vaccinated** | **White British not vaccinated** |
| --- | --- | --- | --- |
| **Level of knowledge** |  |  |  |
| Had not heard about the vaccine prior to invitation | 3 | 6 | 4 |
| Had heard about the vaccine prior to invitation | 4 | 3 | 1 |
| **Information requirements** |  |  |  |
| More information on side-effects | 6 | 3 | 4 |
| Other information on safety | 3 |  | 4 |
| Other information about the vaccine | 7 |  | 2 |
| Other information in general | 7 |  | 5 |
| Information provided in a different format | 4 | 1 |  |
| **Further research** |  |  |  |
| Did very little research | 2 | 5 |  |
| Mind made up before reading information | 3 |  |  |
| Did a lot of research | 2 | 1 | 5 |
| Motivated because of insufficient information | 2 |  | 1 |
| Wanted to confirm decision not to vaccinate | 2 |  | 1 |
| Type of person who questions things | 2 | 1 | 3 |
| **Theme 4 - Preventing HPV-related cancer using means other than vaccination** | **Ethnic minority not vaccinated** | **Ethnic minority vaccinated** | **White British not vaccinated** |
| **Illness prevention informed by CAM and idiosyncratic beliefs** |  |  |  |
| Prefer not to use medicines | 2 | 1 |  |
| Use of methods based on idiosyncratic beliefs or CAM | 3 |  | 2 |
| **Preventing cervical cancer using approaches other than vaccination** |  |  |  |
| Safe sex | 3 |  | 1 |
| Education / discussion | 3 |  | 1 |
| Abstinence from sex before marriage | 5 |  |  |
| Not being promiscuous | 2 |  |  |
| **External forces** |  |  |  |
| External forces | 2 |  |  |

*Note: n* denotes number of participants who mentioned the sub-theme
